# How Effective Is Iron Supplementation During Pregnancy and Childhood in Reducing Anemia Among 6–59 Months Old Children in India?

**DOI:** 10.3389/fpubh.2020.00234

**Published:** 2020-07-07

**Authors:** Monica Steffi Thomas, Anahit Demirchyan, Vahe Khachadourian

**Affiliations:** Gerald and Patricia Turpanjian School of Public Health, American University of Armenia, Yerevan, Armenia

**Keywords:** anemia in children, predictors, iron supplementation, India, National Iron Plus Initiative

## Abstract

**Objectives:** The study sought to identify whether iron and folic acid supplementation of pregnant women and preschool children is associated with child's anemia status and the predictors of anemia among children in India.

**Design:** Secondary data analysis was performed using the National Family Health Survey 4 data. Multivariable logistic regression was used to identify the adjusted associations between child's anemia status and iron supplementation, both during pregnancy and childhood. Also, a model of significant predictors of anemia among children was fitted.

**Setting:** India.

**Participants:** Youngest children (6-59 months) in families.

**Results:** The adjusted association between supplementation during pregnancy and child's anemia status was significant (*p* = 0.010), whereas the adjusted association between supplementation during childhood and child's anemia status was insignificant (*p* = 0.16). The variables independently associated with anemia status of the child included younger age (95% CI 2.67–2.86), child's recent diarrhea (95% CI 1.02–1.14), low birth weight (95% CI 1.17–1.27), current underweight (95% CI 1.14–1.28), diet diversity score (95% CI 0.96–0.98), higher birth order (95% CI 1.01–1.05), mother's current anemia (95% CI 1.68–1.81), months of breastfeeding (95% CI 0.99–1.00), no/primary education (95% CI 1.23–1.35), family's low wealth index (95% CI 1.11–1.23), and backward caste (95% CI 1.04–1.14).

**Conclusions:** The National Iron Plus Initiative strategy of child's iron supplementation should be evaluated to identify the reasons of its ineffectiveness in anemia reduction. In addition, vulnerable groups of children, i.e., children from poor and less educated families and those with low birth weight, higher birth order, and poor nutritional status, should be targeted first with anemia reduction interventions.

## Introduction

### Burden

The prevalence of anemia among children under five was 41.7% worldwide in 2016. In India, this rate was 57.3% (1) and the prevalence of anemia among children 6–59 months of age was 58.5% (2); thus indicating a major public health problem. Compared to other age groups, children under five have a disproportionately higher level of anemia (3). Iron deficiency is considered to be a major cause of anemia in childhood (4). Iron deficiency anemia is associated with a number of other health conditions among children, like behavioral problems, cognitive impairment, stunted growth, and psychomotor development (5–7). Studies from different parts of the world have often found different sets of determinants for iron deficiency anemia, suggesting possible variations in the risk factors across countries.

### Prevention Strategies

Iron supplementation is a part of World Health Organization (WHO) (4) guidelines in combating anemia, which has been successfully implemented in many countries to treat and prevent anemia (8). In 2013, the National Iron Plus Initiative (NIPI) was launched in India to combat the increasing prevalence of anemia (9). It replaced the National Nutritional Anemia Control (NNAC) program which existed since 1991 (10). According to NIPI recommendations, children aged 6–59 months should receive a preparation containing 20 mg elemental iron and 100 mcg folic acid twice a week (9). For pregnant women, the recommendation is using 100 mg elemental iron and 500 mcg folic acid daily for 100 days beginning at 14–16 weeks of gestation (9). It should be noted that the NIPI's iron and folic acid (IFA) supplementation schedule differs in some extent from the WHO recommendations in both the dosage and duration.

### Study Rationale and Aim

Despite the ongoing NIPI program, the prevalence of childhood anemia continues to be alarmingly high in India, and no study has yet evaluated the effectiveness of the NIPI program nationally. The current study aimed to fill in this gap and provide evidence that can be used by policymakers to improve the NIPI program and specifically target the most vulnerable groups of children with anemia reduction interventions. Hence, the study objectives were (1) to identify whether there is difference in anemia rates between those children whose mothers followed the iron supplementation schedule during pregnancy and those children whose mothers did not follow it, (2) to identify whether there is significant difference in anemia rates between those children who follow the iron supplementation schedule and those who do not follow it, and (3) to find the predictors of anemia among 6–59-month-old children in India.

## Methods

### Study Design

Secondary data analysis was performed using the data from the National Family Health Survey 2015–16 (NFHS-4). NFHS-4 was carried out by the International Institute for Population Sciences, Mumbai, under the supervision of the Government of India (2). It was a household survey collecting data on the characteristics of population and their health and nutrition (2). The survey was accompanied with anthropometric measurements of children and testing of adults and young children for blood hemoglobin level, using the HemoCue instrument (11). The NFHS 4 used the HemoCue Hb 201+ instrument because of the rapid results, simplicity of operation, and known precision and accuracy (12). It is based on the cyanomethemoglobin method and thus proven to be “stable and durable in field settings” (13). The NFHS-4 samples were nationally representative and included 601,509 households, 103,525 men (15–54 years), and 699,686 women (15–49 years). The fieldwork for NFHS-4 was carried out from January 2015 to December 2016 (2). The study dataset can be downloaded by registering at the DHS program (14), and the questionnaire can be accessed at the national family health survey website (http://rchiips.org/NFHS/nfhs4.shtml) (15).

### Target Population and Variables

The target population for this study comprised a subsample of the youngest children in surveyed families aged 6–59 months taken from the NFHS-4 dataset. The youngest children in families were chosen, as the information on certain study variables (folic acid and iron supplementation during pregnancy, child's diet diversity, and blood sample given by mother during pregnancy) was available only for them.

The outcome variable was the presence or absence of anemia in a child. The criterion used to determine anemic children was the hemoglobin level <110 grams per liter at the sea level (16). This is the cutoff value for identifying anemia among 6–59 months old children recommended by WHO, which is adopted and used in India as well (13, 17, 18). The exposures of interest were IFA supplementation of mother during pregnancy (whether she was supplemented at least 50 days) and IFA supplementation of the child (at the time of the survey). The covariates and potential confounders considered were age and gender of the child, education of parents, family's wealth index, caste of the household, current anemia of the mother (hemoglobin adjusted for sea level below 120 g/L) (13, 17), blood sample given by the mother during pregnancy (as the only available proxy for pregnancy anemia), mother's young age (<18 years old) at child's birth, child's breastfeeding duration, diet diversity of the child, recent diarrhea in the child, child's undernutrition (stunting, wasting, and underweight), type of household heating and cooking fuel (low-quality biofuel such as straw/shrubs/grass, agricultural crops, and animal dung vs. other fuels), child's birth order, and birth weight.

### Data Analysis

The analysis was done using SPSS 23 statistical software package. Descriptive data analysis of the selected characteristics was done comparing the groups of 6–59-month-old children with and without anemia. The analysis took into account the survey design (clustering and stratification) and applied sampling weights. The statistical significance of the difference between the selected characteristics was measured using chi-square test for categorical variables and student *t*-test for continuous variables. For logistic regression analysis, the categorical variables with more than two categories were transformed into dummy variables. All the variables with *p* < 0.25 in the descriptive analyses were entered into univariate logistic regression analyses (done in complex samples package) with the dependent variable, anemia status of the child. Then, multivariable logistic regression for the weighted data was applied to evaluate the adjusted associations of interest while controlling for all available potential confounders. The large sample size of this study resulted in ample significant associations, therefore, in addition to the statistical significance; an effect size was calculated by comparing the change of the odds ratio of the main association when adding each identified confounder in the multivariable model. Only those categorical variables that produced at least 5.0% change of the odds ratio of the main association were included in the final multivariable logistic regression models. As the last step, a logistic regression model of independent predictors of anemia in 6–59-month-old children was fitted and the model fit evaluated via the Hosmer–Lemeshow goodness-of-fit test and the area under the ROC curve.

### Ethical Review

The study protocol did not require any approval by the Institutional Review Board as it was a secondary analysis of a de-identified dataset.

## Results

### Descriptive Findings

In total, 145,904 last-born 6–59-month-old children were included in the analysis. Among the children, 39.9% were non-anemic (≥11 g/dL), 27.8% mildly anemic (10–10.9 g/dL), 30.7% moderately anemic (7–9.9 g/dL), and 1.6% severely anemic (<7 g/dL).

As Table 1 shows, the sample consisted of 54.7% male and 45.3% female children. Of all the 16 variables analyzed, gender was the only variable that was not statistically significantly related with the anemia status in crude comparisons. Younger age, low birth weight (<2,500 g), recent (within the last 2 weeks) diarrhea, and all the three types of under-nutrition (stunting, wasting, and underweight) were more prevalent in the group of anemic children as compared to those without anemia. The mean dietary diversity score was lower among anemic children than among those without anemia (2.26 vs. 2.45 of the maximum score of 12). Children in the anemic group had higher mean birth order as compared to those in the non-anemic group.

**Table 1:** Descriptive analysis of the selected characteristics among the last born 6–59-month-old children in India by “anemia status” (based on National Family Health Survey 2015–2016 data).

**Characteristic**	**No anemia (*****n*** **=** **58,170)**		**Anemia (*****n*** **=** **87,735)**		***P*-value^**#**^**	**Total sample (*****n*** **=** **145,905)**	
	**Number**	**Percentage**	**Number**	**Percentage**		**Number**	**Percentage**
**CHILD-RELATED VARIABLES**							
**Sex of the child**							
Male	32025	55.1	47980	54.7	0.35	80005	54.8
Female	26144	44.9	39755	45.3		65899	45.2
**Age of the child**							
6–23.9 months old	19193	33.0	44121	50.3	<0.001^*^	63314	43.4
24–41.9 months old	19504	33.5	27804	31.7		47307	32.4
42–60 months old	19473	33.5	15810	18.0		35283	24.2
**Child's recent diarrhea (last two weeks)**							
No	52821	90.9	77452	88.3	<0.001^*^	130274	89.3
Yes, last two weeks	5314	9.1	10230	11.7		15543	10.7
**Birth weight**							
Less than 2.5 kg	17284	35.5	27646	39.7	<0.001^*^	44930	38.0
More than 2.5 kg	31454	64.5	41999	60.3		73453	62.0
**Stunting of child**							
No	13176	22.7	17152	19.6	<0.001^*^	30328	20.9
Yes	44785	77.3	70228	80.4		115012	79.1
**Wasting of child**							
No	10898	18.8	15450	17.6	<0.001^*^	26347	18.1
Yes	47218	81.2	72174	82.4		119392	81.9
**Underweight child**							
No	7483	12.9	9070	10.4	<0.001^*^	16552	11.4
Yes	50478	87.1	78310	89.6		128788	88.6
**Iron supplementation of child (between twelve and fifty-nine months)**							
No	42077	72.7	64490	73.9	0.001^*^	106568	73.4
Yes	15827	27.3	22751	26.1		38578	26.6
**PARENT'S RELATED VARIABLES**							
**Highest educational level of mother**							
No education	13193	22.7	27348	31.2	<0.001^*^	40541	27.8
Primary	7293	12.5	12502	14.3		19796	13.6
Secondary	29219	50.2	39299	44.8		68518	47.0
Higher	8464	14.6	8585	9.8		17049	11.7
**Highest educational level of father**							
No education	1375	13.5	2720	18.2	<0.001^*^	4096	16.3
Primary	1302	12.8	2281	15.2		3583	14.3
Secondary	5735	56.4	8021	53.6		13755	54.7
Higher	1753	17.2	1951	13.0		3704	14.7
**Current anemia of mother (<120 g/dL)**							
No	29455	50.9	31583	36.1	<0.001^*^	61038	42.0
Yes	28461	49.1	55836	63.9		84297	58.0
**IFA supplementation of mother during pregnancy**							
No	14692	32.1	24113	36.6	<0.001^*^	38805	34.7
Yes	31132	67.9	41786	63.4		72918	65.3
**Given blood sample during pregnancy**							
No	5489	14.6	10553	14.6	<0.001^*^	16042	13.2
Yes	44295	89.0	61608	85.4		105903	86.8
**HOUSEHOLD-RELATED VARIABLES**							
**Wealth index**							
Poorest	11275	19.4	22693	25.9	<0.001^*^	33968	23.3
Poorer	11656	20.0	19180	21.9		30836	21.1
Middle	11432	19.7	17593	20.1		29025	19.9
Richer	12398	21.3	15627	17.8		28025	19.2
Richest	11408	19.6	12641	14.4		24049	16.5
**Type of caste or tribe of the household**							
Scheduled caste	11565	21.0	19424	23.1	<0.001^*^	30988	22.3
Scheduled tribe	5065	9.2	9688	11.5		14753	10.6
Other backward class	25473	46.2	38600	45.9		64073	46.0
None of them	13033	23.6	16415	19.5		29448	21.1
**Type of cooking fuel**							
Electricity, LPG, natural gas and biogas	22705	41.0	28210	34.0	<0.001^*^	50916	36.8
Kerosene, coal, lignite, charcoal, wood	24401	44.0	39839	48.0		64241	46.4
Straw/shrubs/grass, agricultural crops, animal dung	8298	15.0	14901	18.0		23199	16.8
**Continuous variables**	**Mean**	**Standard deviation**	**Mean**	**Standard deviation**		**Mean**	**Standard deviation**
**Child-related variables**							
Child's diet diversity score (range: 0–12)	2.4	2.5	2.2	2.3	<0.001^*^	164574	2.3
Child's birth order	2.2	1.3	2.3	1.4	<0.001^*^	145905	2.3
**Parent's related variables**							
Age of mother at first birth (years)	21.2	3.7	20.9	3.5	<0.001^*^	145905	21.0
Months of breastfeeding	23.9	20.1	21.6	18.8	<0.001^*^	145905	22.5

# *p-values from chi-square or Anova test*.

* *Significant values*.

Of mothers of the studied children, 65.3% used IFA supplements during pregnancy, while only 26.6% of the studied children were taking IFA supplements at the time of interview. Mothers and fathers of children in the anemia group were less educated compared to parents of children without anemia. Mothers of children with anemia were younger, suffered from anemia (blood hemoglobin <120 g/dL at the sea level) more frequently, and breastfed for fewer months than mothers of non-anemic children.

Families of anemic children had lower wealth index as compared to families of non-anemic children. Children who belonged to a scheduled caste or tribe or other backward caste had a higher prevalence of anemia. High-quality fuel (electricity, LPG, natural gas, and biogas) usage was more prevalent in the non-anemic group as compared to the anemic group (41.0 vs. 34.0%), while families in the anemic group used lower quality biofuel more frequently (18.0 vs. 15.0%).

### Relation Between Iron Supplementation and Childhood Anemia

Table 2 shows the results of multivariable logistic regression analysis with “anemia status” as the outcome and supplementation during pregnancy as the independent variable controlled for all the available potential confounders, including all the continuous variables regardless of their effect size and those categorical variables that produced over 5.0% change in the odds ratio of the main association when being included in the model. As demonstrated, there is significant independent negative relation between the variables of interest indicating that children of mothers who used IFA supplements for at least 50 days during pregnancy are 10.0% less likely to have anemia.

**Table 2:** Controlled associations between “anemia status of the child” and the two independent variables: “mother's IFA supplementation during pregnancy” and “iron supplementation during childhood” among the last born 6–59-month-old children in India (based on NFHS-4 data).

**Characteristic**	**Odds ratio**	**Confidence interval**		***P*-value**
		**Lower**	**Upper**	
Iron and folic acid supplementation of mother during pregnancy**	0.90	0.83	0.97	0.01n^*^
Iron and folic acid supplementation of child***	0.92	0.82	1.03	0.16

* *Significant value*.

** *This association is controlled for highest educational level of mother, highest educational level of father, wealth index, child's recent diarrhea, cooking fuel types, birth weight of child, IFA supplementation of child, anemia of mother, dietary diversity score, months of breastfeeding, birth order, stunting of child, wasting of child, and blood sample given during pregnancy. Not controlled for caste or tribe of household, underweight child, age of child, and age of mother at first birth, due to small effect size of these variables*.

*** *This association is controlled for age of child, highest educational level of mother, highest educational level of father, wealth index, caste or tribe of household, child's recent diarrhea, cooking fuel types, birth weight of child, IFA supplementation of child, anemia of mother, dietary diversity score, months of breastfeeding, age of mother at first birth, birth order, stunting of child, and wasting of child. Not controlled for child's underweight, due to small effect size of this variable*.

The multivariable logistic regression with “anemia status” as the outcome and child's supplementation as the independent variable, controlled for all the available potential confounders, including all the continuous variables regardless of their effect size and those categorical variables that produced over 5.0% change in the odds ratio of the main association, found no significant relation between child's IFA supplementation and his/her anemia status (Table 2).

### Predictors of Childhood Anemia

The fitted multivariable logistic regression model (Table 3) identified 11 independent predictors of anemia among 6–59-month-old children in India. Among these, age was a protective factor for anemia with 6–23-month-old children having 2.77 times higher odds and 24–41-month-old children 1.73 times higher odds of suffering from anemia compared to the oldest age group of 42–59-month-old children. An episode of recent diarrhea (within last 2 weeks) increased the odds of the child being anemic by 8.0%. Children whose birth weight was <2.5 kg had 22.0% increased odds of being anemic as compared to children whose birth weight was more than 2.5 kg. An underweight child had a 21.0% increased chance of being anemic as compared to a normal-weight child. Each one-unit increase of diet diversity score decreased the risk of the child's anemia by 3.0%. With each unit increase in the birth order, a child was at a 3.0% increased likelihood of being anemic. Current anemia of the mother increased the likelihood of the child being anemic by 74.0%. With every 1-month increase in breastfeeding duration, the odds of a child being anemic decreased by 1.0%. Mothers with no or primary education had 29.0% higher odds for their child being anemic as compared to mothers with secondary or higher education. Children from households with poor and middle wealth index had, respectively, 1.17 and 1.11 times higher odds of being anemic as compared to children from households with rich wealth index. Scheduled caste, scheduled tribe, and other backward caste households had 9.0% higher odds of the child being anemic as compared to any other caste households.

**Table 3:** Multivariate logistic regression model of anemia predictors among the last born 6–59-month-old children in India (based on NFHS-4 data; valid *n* = 49,162).

**Characteristic**	**Odds ratio**	**Confidence interval**		***P-*value**
		**lower**	**upper**	
**Age of the child (months)**				
6–23.9 months	2.77	2.67	2.86	<0.001^*^
24–41.9 months	1.73	1.67	1.79	<0.001^*^
42–60 months	1.00	Reference		
**Child's recent diarrhea (last two weeks)**	1.08	1.02	1.14	0.007^*^
**Low birth weight of the child (<2,500 g)**	1.22	1.17	1.27	<0.001^*^
**Underweight child**	1.21	1.14	1.28	<0.001^*^
**Diet diversity score**	0.97	0.96	0.98	<0.001^*^
**Child's birth order**	1.03	1.01	1.05	<0.001^*^
**Current anemia of mother (<120 g/dl)**	1.74	1.68	1.81	<0.001^*^
**Months of breastfeeding**	0.99	0.99	1.00	0.040
**Highest educational level of the mother**				
No education or Primary	1.29	1.23	1.35	<0.001^*^
Secondary and Higher	1.00	Reference		
**Wealth index**				
Poor	1.17	1.11	1.23	<0.001^*^
Middle	1.11	1.05	1.17	<0.001^*^
Rich	1.00	Reference		
**Type of caste or tribe of the household**				
Scheduled caste, scheduled tribe, other backward class	1.09	1.04	1.14	<0.001^*^
Any other class	1.00	Reference		

*Hosmer and Lemeshow test: 0.21*.

*Area under the ROC curve: 0.62*.

* *Significant values*.

## Discussion

### Prevalence and Dynamics of Childhood Anemia

According to this study results, the prevalence of anemia among 6–59-month-old children in India was 60.1%, which reflects a significant decrease from the prevalence of 69.4% found by NFHS-3 in 2005–06 (19). This decrease can be due to the measures undertaken in the scope of the NIPI Program. However, the observed decrease is far from the ultimate goal of the program to combat childhood anemia in the country. Moreover, the current rate of anemia among under-five children in India still represents a “severe public health problem” according to WHO criteria (20). It is comparable to that in Pakistan (59.0%) and is considerably higher than in Bangladesh (40.0%) and the world average (41.7%) (21). Anemia rates are equally high among both genders, which is consistent with a study conducted using the Ghana demographic and health survey that found no significant difference in anemia prevalence among boys and girls (22).

### Iron Supplementation and Childhood Anemia

This study found no significant association between IFA supplementation during preschool years and child's anemia status, even though in many countries a significant association has been found (23, 24). Potential explanations for this could include compliance and logistics-related challenges in the NIPI program (9) as well as other shortcomings that this program might have inherited from the prior NNAC program. As to the latter, a study evaluating the NNAC program in Andhra Pradesh found that only 19.0% of the pregnant women and 1.0% of the child beneficiaries had received their supplementations (25). The main reasons for the poor coverage included inadequate and irregular supplies of the supplements and incomplete registration of beneficiaries by the health centers (25). The chemical analysis of the supplements also found out that 30.0% of them had less than recommended iron levels and none of them had the recommended folic acid levels (25). A study that evaluated the NNAC program in the Dharwad *taluk* also found an issue of irregular supply of the supplements (26). A cross-sectional study evaluating the compliance of the NIPI program among a group of reproductive aged (15–49 years) females in Puducherry found that only 45.7% of them were receiving the supplements under the NIPI program (27). Among the women who received supplementation, only 61% reported compliance (27). A qualitative study in Odisha showed the necessity of monitoring and evaluation to strengthen the implementation of the NIPI program (28). Another possible reason of ineffectiveness of iron supplementation in reducing anemia among Indian children could be an underlying multiple micronutrient deficiency, as found in a study done among Mexican preschoolers (29).

IFA supplementation of the mother during pregnancy is found to have a preventive effect on anemia status of the child in this study. According to a number of studies, the mechanism of this effect is that routine supplementation of the mother during pregnancy prevents a bunch of conditions associated with increased risk of childhood anemia, including maternal anemia (30–32), low birth weight (33), and preterm birth (34–37). An interesting finding of the study was that during the analysis, the initial controlled association between pregnancy supplementation and child's anemia was positive, meaning that pregnancy supplementation acted as a “risk factor” for child's anemia. As there was no available data on woman's anemia status during pregnancy, the research team suspected that this finding might be the reflection of a practice of prescribing supplements specifically to those pregnant women with known anemia. In this case, pregnancy supplementation could indirectly point out the women with anemia during pregnancy. This suspicion was confirmed by the fact that the relation between pregnancy supplementation and childhood anemia became protective after including in the model “giving blood sample during pregnancy,” the only available proxy variable for pregnancy anemia. The practical implication of this finding is the need to reinforce the compliance with the recommended pattern of universal preventive prescription of IFA supplements to all pregnant women regardless of their anemia status. Another inference that can be made from this finding is that the actual protective effect of iron supplementation during pregnancy on child's likelihood of developing anemia could be even stronger if the data on pregnancy anemia status was available and controlled for.

### Predictors of Childhood Anemia

Literature reports the following common determinants of childhood anemia: age of the child (30, 38–41), exclusive breastfeeding duration (31), literacy of parents (31, 40), household income (31, 32, 38, 40), caste of the household (42, 43), geographical location (32, 40), anemia of mother (30–32), nutritional status of the child (30–32, 38, 41), and diarrhea (31). This study found 11 independent predictors of anemia among under-five children. Child-related predictors included younger age of the child, child's recent diarrhea, low birth weight, underweight, low diet diversity score, and higher birth order. In a number of studies, as in this study, the age group of 24 months and below was strongly associated with anemia in the child (30, 38). This is due to the high-iron requirements of the child because of high growth speed during this age (44), often combined with poor dietary diversity resulting in consumption of a diet which doesn't include iron rich foods (45). An episode of recent diarrhea (within the past 2 weeks) in a child was found to be positively associated with child's anemia status in other studies as well (31, 46). According to these studies, poor absorption of nutrients and decreased food consumption of the child during diarrhea could be among the underlying mechanisms of this association (31, 47, 48). The findings of this study on child's low birth weight and current underweight predicting anemia are also consistent with the literature. A study conducted in a rural area in China found that anemia was significantly associated with low birth weight and malnutrition (underweight, stunting, and wasting) (33). The mechanism underlying the relation between low birth weight and anemia is that hemoglobin and ferritin concentrations are found to be positively correlated to the birth weight of a child (49, 50). Poor dietary diversity is also a known predictor of anemia (51, 52), due to its direct relation with inadequate micronutrient intake (52). Higher birth order is another factor positively associated with anemia in a child not only in this study, but in a number of other studies (53, 54).

In this study, the identified predictors related to maternal factors included current anemia of mother, months of breastfeeding, and educational level of mother. A study conducted in Timor Leste found that the severity of anemia of mothers was directly correlated to the severity of anemia in children (31), showing that anemia of mother is a predictor of anemia status of children. This is due to the correlation of maternal hemoglobin with cord blood hemoglobin concentrations and breast milk iron levels (55, 56). Consistent with this study, a study conducted in Eastern Cuba showed that lack of breastfeeding is a predictor of anemia (57). The underlying mechanism is the presence of highly bioavailable iron in breast milk (58). The finding that children of mothers with higher education are at lower risk of anemia is again consistent with other studies (40). This is due to ensuring better dietary practices of children, including consumption of iron rich foods, by those mothers having higher educational level (59).

The household-related predictors included wealth index and type of caste or tribe of the household. The wealth index of the household was associated with anemia status of the child with a dose-response relationship, as the poorest households had the highest risk and the middle households had higher risk of the child being anemic as compared to the rich households. This relationship is seen in other countries as well, which is due to better nutrition and health services to children from richer households (60). Children of scheduled caste, scheduled tribe, and other backward castes have increased risk of anemia both in this and other (42) studies, which is due to substandard living conditions, unfavorable behaviors (maternal smoking, poor dietary habits), and inefficient healthcare use among these castes (42).

### Study Strengths and Limitations

This study had a number of limitations. Similar to most secondary data analysis studies, the current study was limited to the variables covered in the NFHS-4 survey and did not have any control over the data collection process (61). The NFHS-4 questionnaire collected no information on the dosage or duration of the child's supplementation. The only question on this matter was whether the child was taking supplements currently, which might have introduced an instrumentation error, making it impossible to identify children who received supplements according to the existing recommendations. For women's supplementation, we only took those women who had taken the supplements for at least 50 days out of the 100-day criteria. The NFHS-4 survey used the HemoCue Hb 201+ instrument which has a sensitivity of 75–91% and specificity of 88–100% for anemia (62). This instrument is mainly used for screening of anemia and requires a full blood count for confirmation (62). The test results do not differentiate types of anemia. Data on the anemia status of mothers during pregnancy was not available, and a proxy variable of “whether blood sample was collected during pregnancy” had to be used instead. Also, the study lacked information on the actual content of supplements, their quality and availability, and whether they were prescribed in accordance with the current recommendations of the NIPI program.

Despite these limitations, the study produced very interesting results that could be used for developing better targeted strategies to reduce childhood anemia in India. This study was the first attempt of measuring the impact of the NIPI program on anemia status of children using an impressive nationwide sample and an analytical approach that applied sampling weights, thus making the findings nationally representative. Also, the study results delineated areas for further research, including investigation of the lack of relation between child's iron supplementation and anemia status, investigation of the content and quality of the supplements for children, the extent of compliance with the recommended regimen of administration and the actual coverage of pregnant women and preschool children with the supplements, and investigation of the possible relation between pregnancy anemia and prescription of IFA supplements during pregnancy. The questionnaire of NFHS-5 needs to be revised to include questions on child supplementation and maternal anemia to get a better idea of the problem in future.

### Recommendations

This study identified areas that can be of short- and long-term targets for the Indian government. The short-term objectives include addressing the challenges of NIPI in every state and prioritizing anemia as a major public health problem. A study conducted in India showed that the “estimated yearly costs of iron deficiency anemia in 6–59 months old children amount to intangible costs of 8.3 million disability adjusted life years and production losses of 24,001 million USD, equal to 1.3% of gross domestic product” (63). A randomized, double-blinded study done among Indian school children showed that iron-fortified flour significantly improved body iron stores and reduced iron deficiency anemia (64). A study conducted in 10 developing countries showed that long-term iron fortification programs have a median cost benefit ratio of 6:1, which becomes 36:1 if the impact on cognitive improvements are also included (65), clearly showing that this is an efficient solution. Thus, in the long term, if anemia continues to remain a prevalent issue, food fortification can be another option for combating it. Based on this study findings, the vulnerable groups of children, i.e., children from poor and less educated families, those with low birth weight, higher birth order, and poor nutritional status, should be targeted first with anemia reduction interventions in India.

The study also measured the effect of the nationwide iron and folic acid supplementation program in India on children's anemia status while the NIPI program is still ongoing, thus creating a knowledge base for refining the program. Therefore, our results may contribute to learning and decision-making through the course of the program implementation to achieve better performance, compliance, and hence, better outcomes.

## Data Availability Statement

The datasets generated for this study are available on request to the corresponding author.

## Author Contributions

MT formulated the research question, analyzed the data, and had primary responsibility for the final content. AD, VK, and MT designed and carried out the research and wrote the paper. All authors contributed to the article and approved the submitted version.

## Conflict of Interest

The authors declare that the research was conducted in the absence of any commercial or financial relationships that could be construed as a potential conflict of interest.

## Abbreviations

LPG: liquefied petroleum gas
NFHS: National Family Health Survey
NIPI: National Iron Plus Initiative
NNAC: National Nutritional Anemia Control
ROC: receiver operating characteristic
SPSS: Statistical Package for the Social Sciences
USD: United States Dollar
WHO: World Health Organization.
